# Non-radioactive 2-deoxy-2-fluoro-D-glucose inhibits glucose uptake in xenograft tumours and sensitizes HeLa cells to doxorubicin *in vitro*

**DOI:** 10.1371/journal.pone.0187584

**Published:** 2017-11-02

**Authors:** Sarah Niccoli, Douglas R. Boreham, Christopher P. Phenix, Simon J. Lees

**Affiliations:** 1 Medical Sciences, Lakehead University Faculty of Medicine, Thunder Bay, Ontario, Canada; 2 Medical Sciences Division, Northern Ontario School of Medicine, Thunder Bay, Ontario, Canada; 3 Chemistry, University of Saskatchewan, Saskatoon, Saskatchewan, Canada; 4 Thunder Bay Regional Health Research Institute, Thunder Bay, Ontario, Canada; 5 Biology, Lakehead University, Thunder Bay, Ontario, Canada; Instituto Nacional de Cardiologia, MEXICO

## Abstract

A glucose analog called 2-deoxy-D-glucose (2DG) has been successfully used to sensitize cancer cells to ROS-inducing cancer treatments such as ionizing radiation, through the inhibition of glycolysis. However, the use of 2DG can be limited by several factors such as availability, non-specific cytotoxicity, and chemoresistance under hypoxic conditions. The purpose of this study was to investigate the use of non-radioactive 2-deoxy-2-fluoro-D-glucose (^19^FDG), a drug that potentially addresses current limitations of 2DG. The effectiveness of using either 2DG or ^19^FDG in combination with doxorubicin (Dox) in HeLa cells was determined in both normoxia and hypoxia. We have also shown that under both oxygen conditions, ^19^FDG-treated cells produce less lactate than 2DG-treated cells, an important finding that suggests improved inhibition of glycolysis, the preferential pathway for cancerous cells. When used in combination with Dox, we have demonstrated a significant decrease in the number of viable cells, with the effect of ^19^FDG remaining stable across both normoxic and hypoxic conditions. Moreover, the assessment of apoptosis and necrosis revealed that ^19^FDG maintained its ability to sensitize HeLa cells to Dox in hypoxia, but 2DG was only effective under normoxic conditions. The retained effectiveness of ^19^FDG in combination with Dox under hypoxic conditions, suggests that ^19^FDG may be efficacious for sensitizing hypoxic regions of solid tumour masses. Importantly, the ability of ^19^FDG to inhibit glucose uptake *in vivo* was also confirmed using positron emission tomography (PET) of xenograft tumours. The results displayed here suggest ^19^FDG is a promising combination therapy, which may lead to decreased ROS scavenging via glycolysis, and enhanced treatment success.

## Introduction

For over 80 years it has been noted that cancer cells demonstrate altered metabolism when compared to untransformed cells. Malignant cells, through a phenomenon termed the Warburg effect display significantly elevated use of glucose as an energy source even in the presence of oxygen, which would typically facilitate oxidative phosphorylation. This elevation may be caused by several factors such as mitochondrial defects [1, 2], hypoxic conditions [3, 4], or an alteration in the expression of certain metabolic enzymes [5, 6]. These conditions may result in cancerous cells producing mainly lactate as an end product of glycolysis. Not only does this work to acidify the microenvironment of the tumour, promoting the growth of an aggressive phenotype that can survive these conditions, it also decreases the amount of pyruvate being produced. Pyruvate is a potent antioxidant, and without it, the balance of oxidative stress is disturbed, resulting in a pro-oxidative state [7–9].

Although it is widely understood that an imbalance of oxidative stress is damaging to healthy cells [10, 11], cancerous cells have developed protective mechanisms to combat this intrinsic pro-oxidation. An increase in glycolysis and pentose phosphate cycle activity in cancerous cells, accompanied by only slightly reduced rates of respiration, result in aggressive cells that are faster growing and have induced protective mechanisms against reactive oxygen species (ROS) [12]. Additionally, under hypoxic environments, often present in tumours, oxidative phosphorylation is diminished, further increasing the burden on glucose consumption [13–15] (reviewed in [7, 16–18]).

The importance of increased glucose uptake for the survival and proliferation of cancerous cells has therefore become the focus of many treatment developments. Several compounds have been found to modulate glucose metabolism in cells [19–22]. One such compound is 2-deoxy-D-glucose (2DG), a competitive inhibitor of glucose metabolism that is taken up by glucose transporters and phosphorylated to 2DG-6-phosphate (2DG6P). At this stage, the 2DG6P cannot be further metabolized and accumulates, inhibiting both phosphoglucose isomerase and hexokinase, effectively halting the process of glycolysis at multiple stages [23, 24]. Many studies have been completed testing the efficacy of 2DG [25–31], and clinical trials have been initiated for multiple cancers but have been terminated due to unavailability of the drug produced in good manufacturing practices labs intended for human use, and low recruitment (Clinicaltrial.gov Identifiers: NCT00247403 and NCT00633087). Of these studies, some have shown that on its own, 2DG is not sufficient to significantly inhibit tumour cell growth [25, 28, 29]. Several other limitations of 2DG have been discovered such as its lack of effect under hypoxic conditions [32], and its possible non-specific cytotoxicity to other tissues through interference of N-linked glycosylation of glycoproteins [30]. It is for these reasons that we propose the use of the non-radioactive analog 2-deoxy-2-fluoro-D-glucose (^19^FDG). ^19^FDG accumulates in cells through the same mechanism as 2DG [33] yet has been shown to be a more potent glycolytic inhibitor than 2DG, and maintains its effectiveness under hypoxic conditions [34]. Another significant advantage of ^19^FDG over 2DG is the fact that ^18^F-FDG, the radiolabeled version of ^19^FDG, is already an established tool for imaging glycolysis. As a result, we can use the radiotracer in conjunction with Positron Emission Tomography (PET) to image inhibition of ^18^F-FDG uptake in tumours following ^19^FDG treatment to guide dosing, evaluate the effect of ^19^FDG treatment on non-cancerous tissues, and monitor treatment success. There is minimal toxicity information for ^19^FDG and any observed adverse effects from its radioactive counterpart, ^18^F-FDG, are related to the exposure to radioactivity [35, 36]. It is reasonable to expect the non-radioactive ^19^FDG to display similar toxicity as its analog 2DG, which has been safely given to humans at oral doses of up to 250mg/kg [37].

Although glycolytic inhibition is an important first step to increasing cell death, cells with functioning mitochondria would be able to use other sources such as fatty acids and amino acids as energy to survive [7, 16, 17]. In these cases, further treatment with other anticancer drugs would likely act to increase the detrimental effects on the tumour. Additionally, glycolytic inhibition will lead to a decrease in ROS scavenging [12], so the use of a ROS producer such as Doxorubicin (Dox) will act as a further stressor on the cancer cells.

The purpose of the present study was two-fold. First we sought to investigate the effectiveness of ^19^FDG, compared to 2DG, when used synergistically with Dox *in vitro*. To do this, we utilized the HeLa cervical carcinoma cell culture model in both normoxic and hypoxic conditions and determined the ability of the treatments to inhibit glycolysis, decrease cell viability, and induce apoptosis. Second, we sought to determine the ability of ^19^FDG to inhibit tumour glucose uptake *in vivo*. To do this we utilized the MDA-MB-231 breast carcinoma tumour xenograft model and analyzed tumour glucose uptake using PET imaging. We demonstrate that compared to 2DG, ^19^FDG more effectively inhibits glycolysis, decreases cell viability, and induces apoptosis *in vitro* under both normoxic and hypoxic conditions in conjunction with Dox. Moreover, we also demonstrate that ^19^FDG inhibits tumour glucose uptake *in vivo*.

## Materials and methods

### Cell culture

HeLa (human cervical carcinoma) cells, obtained from the American Type Culture Collection, and MDA-MB-231 (human breast adenocarcinoma) cells, a kind gift from Dr. Giulio Francia at the University of Texas at El Paso, were maintained in 150cm^3^ flasks in a humidified incubator at 37°C, 5% CO_2_, and 20% O_2_. Cells were cultured in High Glucose Dulbecco’s Modified Eagle’s Medium (DMEM) supplemented with either 10% fetal bovine serum (FBS) or fetal clone III serum (FCIII), 1mM sodium pyruvate, 1% penicillin/streptomycin solution (100 U/ml penicillin, 100 μg/ml streptomycin), and 0.05mg/ml gentamicin sulfate.

Hypoxic conditions were created using a Coy Laboratory Products Inc. humidified hypoxic glove box at 1% O_2_. All treatments for hypoxic conditions were made with media that had been deoxygenated in cell culture plates in the chamber for one hour.

### Lactate production

HeLa cells were seeded at a density of 10^4^ cells per well in 24 well plates and grown overnight, after which media was changed to fresh media containing 2.5, 5, 10 or 20mM 2DG (Sigma, D6134) or ^19^FDG (purchased, Sigma F5006). Treatment proceeded for 24 hours under either normoxic or hypoxic conditions. Lactate production was measured from the cell culture media using the Lactate Assay Kit I from Eton Biosciences Inc. as per manufacturer’s protocol. Briefly, 50μl of media was added to the wells of a 96-well plate, followed by 50μl of L-lactate assay solution. The plate was sealed and incubated at 37°C, followed by the addition of 50μl 0.5M acetic acid. The absorbance was read at 490nm, and results were normalized to the amount of protein from the cells.

### Protein assay

To quantify the amount of protein in each cell culture sample for normalization purposes, *DC* Protein Assay kits were used as per manufacturer’s protocol. Briefly, 5μL of sample was loaded into a 96-well plate followed by 25μL of solution A’ and 200μL reagent B. Serial dilutions of Bovine Serum Albumin (BSA, Bio-Rad, 500–0007) beginning at 1.45mg/ml were used to form a standard curve. Absorbance was read at 750nm.

### MTT cell viability assay

HeLa cells were seeded at a density of 8500 cells per well in 96 well plates and grown overnight, after which media was changed to fresh media containing either purchased 10mM 2DG or ^19^FDG, Dox at a dose of 1μg/ml, or a combination of these treatments. Initial treatment stocks were reconstituted or diluted in cell culture grade water before being added directly to the cell culture media. Treatment proceeded for 24 hours under either normoxic or hypoxic conditions, and MTT reagent (5mg/ml thiazolyl blue tetrazolium bromide) was added into the wells at a dilution of 1:10 for the final 3 hours of treatment. Media was then removed from the wells and 100μl dimethyl sulfoxide (DMSO) was added to each well. The plate was incubated protected from light with mild agitation for 10 minutes, and absorbance was read at 540nm.

### Chemical synthesis of 2-deoxy-2-fluoro-D-glucose

^19^FDG was synthesized in the laboratory of Dr. Christopher Phenix as per previous literature [38]. The NMR spectra of the prepared compound were found to be same as those reported in the literatures [38, 39].^1^H NMR (500 MHz, D_2_O)^2^: δ 5.40 (br-d, 0.40H, *J* = 3.8 Hz), 4.86 (dd, 0.60H, *J* = 2.5, 7.6 Hz), 4.37 (ddd, 0.40H, *J* = 3.8, 9.6 Hz, 51.4 Hz), 4.06 (ddd, 0.60H, *J* = 7.6, 9.3, 52.4 Hz), 3.94 (ddd, 0.40H, *J* = 9.3, 9.6, 15.4 Hz), 3.87 (br-d, 0.60H, *J* = 12.2 Hz), 3.82 (dt, 0.60H, *J* = 5.2, 9.6 Hz), 3.77 (ddd, 0.40H, *J* = 9.3, 9.6, 15.1 Hz), 3.73 (dd, 0.60H, *J* = 5.2, 12.7 Hz), 3.69 (dd, 0.40H, *J* = 5.2, 12.2 Hz), 3.68 (dd, 0.40H, *J* = 5.2, 12.7 Hz), 3.49–3.39 (m, 1.60H); ^13^C NMR (125.7 MHz, D_2_O)^1^: δ 93.4 (d, *J* = 22.6 Hz), 92.7 (d, *J* = 182.3 Hz), 90.1 (d, *J* = 186.0 Hz), 89.5 *J* = 21.4 Hz), 75.9 (s), 73.9 (d, *J* = 17.6 Hz), 71.1 (s), 70.5 (d, *J* = 17.6 Hz), 69.1 (d, *J* = 8.8 Hz), 68.5 (d, *J* = 7.5 Hz), 60.1 (s), 60.2 (s). Additional testing (results not shown) was performed in order to confirm the results in the MTT assay using purchased ^19^FDG compared to the lab-synthesized ^19^FDG were the same before continuing use of the lab-synthesized ^19^FDG for further experiments.

### Annexin V-FITC assay

HeLa cells were seeded at a density of 10^5^ cells per well in 6 well plates and grown overnight, after which media was changed to fresh media containing 10mM 2DG or ^19^FDG, 1μg/ml Dox, or a combination of these treatments. Treatment proceeded for 12 hours under either normoxic or hypoxic conditions, after which wells were washed with phosphate buffered saline (PBS) and trypsinized. Neutralized samples were moved into 1.5ml tubes that had been pre-coated at 4°C with 1% BSA/PBS. The ApoScreen^™^ Annexin V-FITC Kit (SouthernBiotech) was then used as per manufacturer’s protocol to observe annexin V and propidium iodide staining with the BD FACSCalibur flow cytometer. BD CellQuest Pro was used for analysis.

### Tumour induction

Female 6 week old athymic nude mice were purchased from Charles River Laboratories (Wilmington, MA). All mice were housed in a clean room at the Lakehead University Animal Care Facility in a HEPA-filtered Innovive^**®**^ Innorack^**®**^ IVC system, and handled in a Biological Safety Cabinet. All experimental procedures were approved by the Animal Care Committee of Lakehead University in line with the recommendations of the Canadian Council on Animal Care (CCAC). All efforts were made to minimize suffering, and analgesics were available if required however they were not needed. Mice were euthanized under deep surgical plane isoflurane anaesthesia by removal of the heart. Mice were allowed one week to acclimate prior to beginning experiments, and allowed free access to sterile food and water. For tumour induction and measurements, mice were anesthetized using isoflurane. Tumour cells were inoculated into the right flank of the mice by subcutaneous (s.c.) injection of 0.1 mL aliquots of serum-free media containing 5 x 10^6^ MDA-MB-231 cells using 27-gauge needles.

### Tumour measurements

Mice were observed daily. Once tumours were visible by eye, mouse weight was monitored closely and body weights were recorded at least twice per week. Tumours were measured once per week using callipers, and volume in mm^3^ was calculated using (length x width x depth) x 0.5235. Tumour measures and body weights, as well as body conditioning scores, were used to determine humane endpoints.

### Drug administration and imaging

Tumours were grown for 3–4 weeks before glucose uptake was confirmed using PET on the Sofie Biosciences G4 PET/X-ray system. An initial ^18^F-FDG PET scan was performed on each mouse to ensure the tumours were actively taking up glucose. Mice were deprived of food 5 hours prior to the scan in order to obtain a clear image with low background. All mice were given a 300μl intraperitoneal (i.p.) injection of saline as an injection control, as well as an i.p. injection of ~20 μCi of radioactive FDG (^18^F-FDG, a kind gift from the Thunder Bay Regional Health Sciences Centre) in saline 1 hour before the scan. The precise dose of ^18^F-FDG was determined using a dose calibrator. Mice were kept under isoflurane anaesthesia during injections and during the 10 minute scan. Maximum standardized uptake value (SUVmax) for each tumour was measured using VivoQuant each week. Once tumour glucose uptake was confirmed (SUVmax of at least 0.75), the mice were divided into treatment groups. One week following group assignment another PET scan was performed. 2 hours before the ^18^F-FDG PET scan, mice in the control group (n = 2) were given 300μl saline i.p. and mice in the ^19^FDG group (n = 3) were given 220 mg/kg ^19^FDG i.p. in saline.

### Statistics

Data are presented as mean ± SEM. Sample sizes are indicated for each measurement in the figure legends. For cell culture studies, comparisons between groups were done using a Two-Way ANOVA and the Fisher’s LSD post-hoc analysis, or a t-test (SigmaStat software, Systat, Chicago, IL). For PET imaging data, comparisons between groups were done using a t-test. Significance was accepted at p≤0.05.

## Results

While 2DG has been shown to successfully inhibit glycolysis in numerous cancer cell types, there are many limitations to the use of this glucose analog for therapy purposes. Here, we directly compare the effects of 2DG and a second glucose analog, ^19^FDG, in their ability to inhibit glycolysis, decrease cell viability, and increase cell death in HeLa cells. Importantly, the ability to inhibit glucose uptake is not limited to cells *in vitro*. We also verified that ^19^FDG is an effective inhibitor of tumour glucose uptake *in vivo* in a xenograft tumour model.

### ^19^FDG is a more effective inhibitor of glycolysis than 2DG

**Fig 1:**
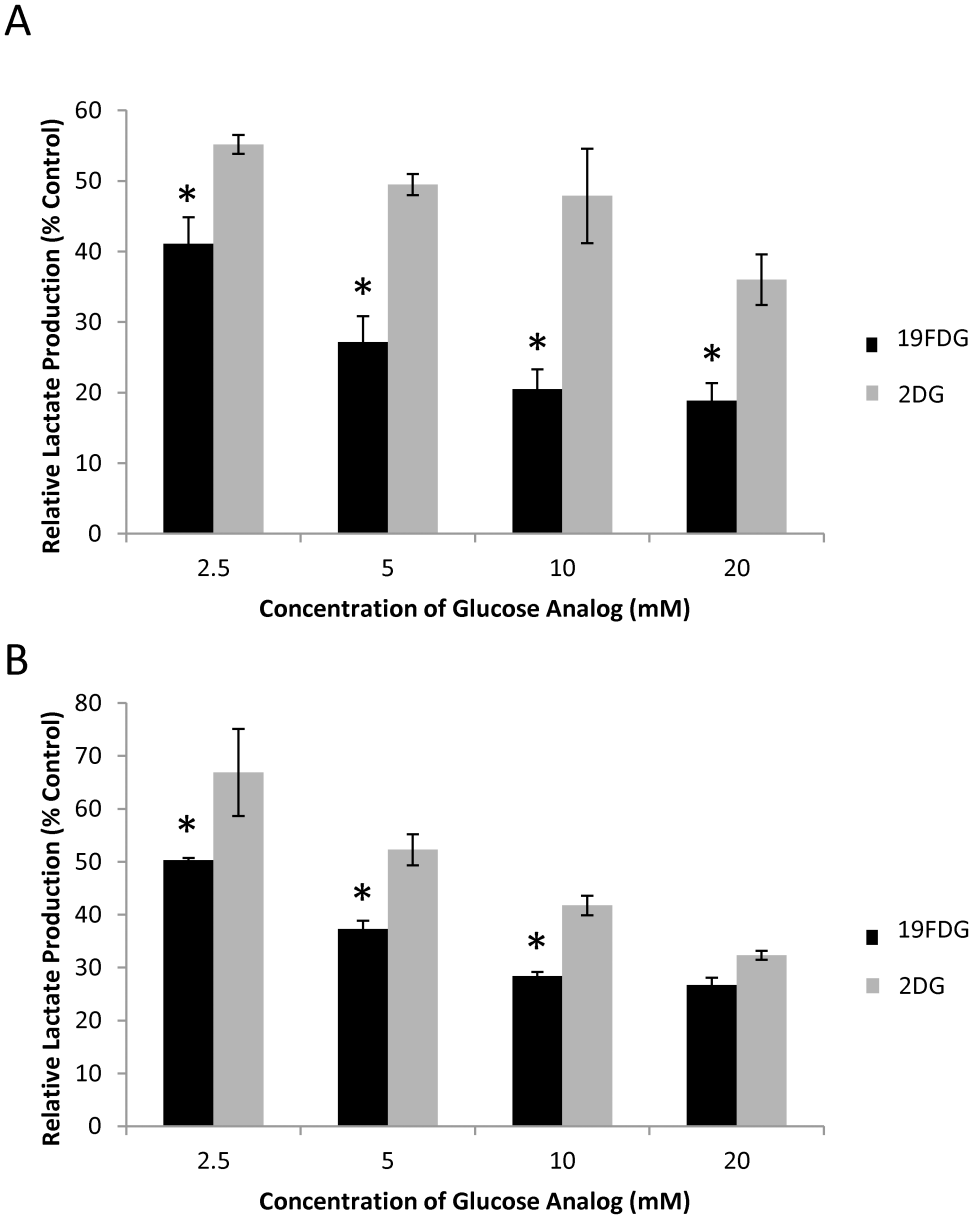
Lactate production from HeLa cells exposed to 2-deoxy-D-glucose (2DG) or non-radioactive 2-deoxy-2-fluoro-D-glucose (^19^FDG) under either normoxic or hypoxic (1% O_2_) conditions. HeLa cells were treated with different doses of 2DG or ^19^FDG for 24 hours under either normoxia (A) or hypoxia (B), and lactate production was measured from the cell culture media. Lactate production was normalized to the amount of protein from the corresponding cells. n = 3, * denotes significance from 2DG within the same glucose analog concentration, p≤0.05. All doses of both analogs significantly decreased lactate production of cells compared to untreated cells.

In order to assess which concentration of glucose analog was most effective for glycolytic inhibition, a dose response curve of each analog was performed measuring lactate production, as this is the preferred glycolytic product of cancerous cells (Fig 1). It was found that under both hypoxic and normoxic conditions, both 2DG and ^19^FDG significantly decreased lactate production of cells compared to untreated control cells. Although each analog displayed a similar trend of decreased lactate production as the concentration of analog increased, ^19^FDG significantly decreased lactate production compared to 2DG at all four doses under normoxic conditions, and at the doses of 2.5, 5, and 10mM under hypoxic conditions. Compared to 2DG, ^19^FDG demonstrated an 25.5%, 45.1%, 57.2%, and 47.6% less lactate production as a result of 2.5, 5, 10, and 20 mM analog, respectively, under normoxic conditions. Based on these results, 10mM was chosen as the glucose analog dose moving forward with the *in vitro* portion of these studies. The decision was based on the finding that very little benefit was observed in glycolysis inhibition by increasing the concentration of ^19^FDG from 10 to 20 mM.

### ^19^FDG maintains its efficacy as an adjuvant treatment in hypoxia

**Fig 2:**
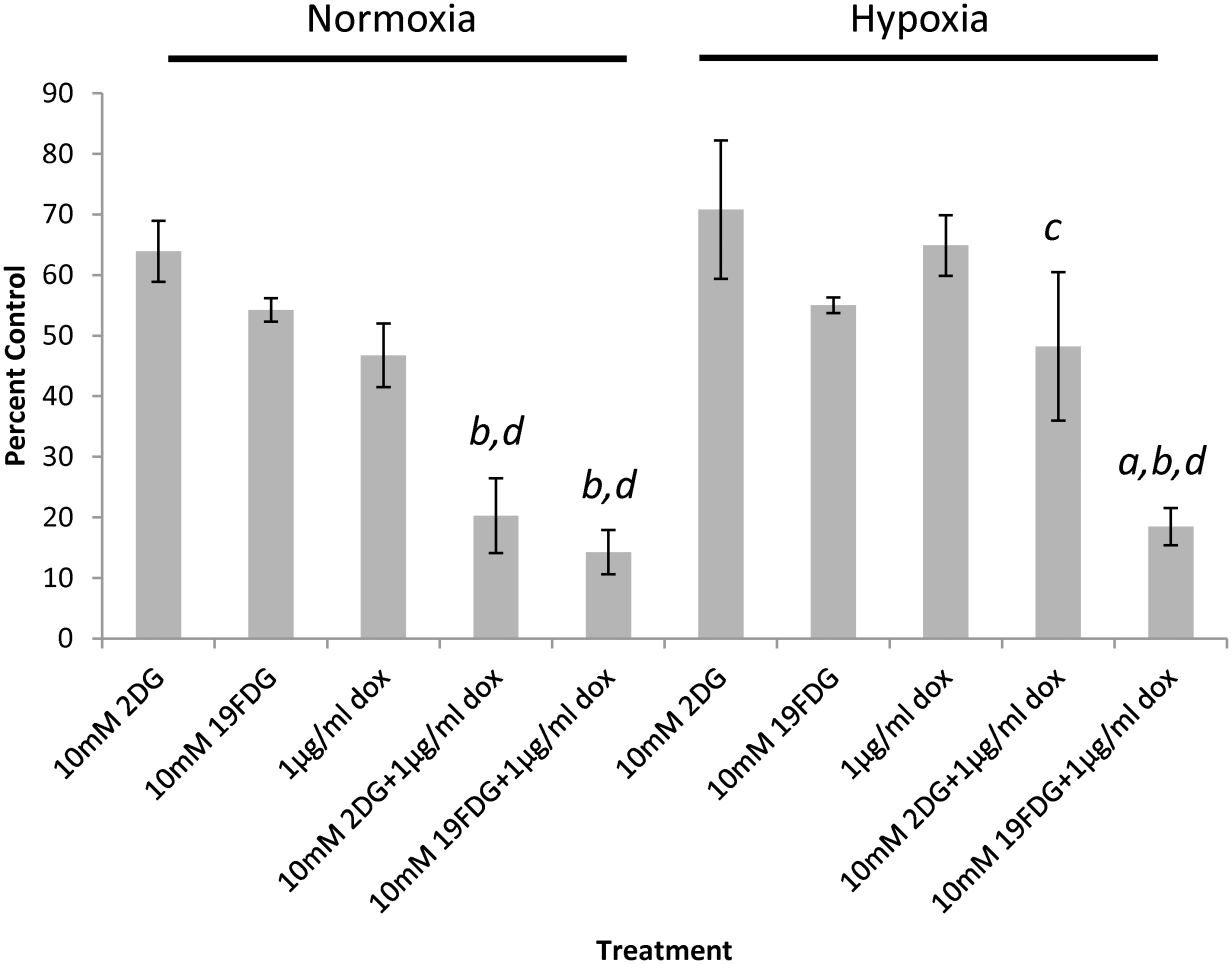
Measurement of HeLa cell viability following combination treatment. MTT was used to measure cell viability 24 hours after each of several treatment conditions; 2-deoxy-D-glucose (2DG), non-radioactive 2-deoxy-2-fluoro-D-glucose (^19^FDG), or 1μg/ml doxorubicin alone or in combination, under normoxia or hypoxia. OD readings are presented as a percentage of the OD from untreated control cells. n = 4. *a*, denotes significance from Dox+2DG, *b* denotes significance from Dox alone, *c* denotes significance from normoxia within that treatment, and *d* denotes significance from 2DG and ^19^FDG alone. p≤0.05.

Doxorubicin is a mitochondrial electron transport chain modulator that promotes ROS production [40]. It has been frequently used in studies and is a standard chemotherapeutic used both on its own and in conjunction with other treatments [41–45], yet it has been rendered ineffective in some cases due to drug-resistant cancer cells. It was therefore of interest to determine whether the use of the glucose analogs could sensitize the cancer cells to Dox treatment (Fig 2). Several Dox doses were tested in order to determine the lowest effective dose (data not shown). The MTT cell viability assay revealed that with a Dox dose of 1μg/ml, the addition of either glucose analog significantly decreased cell viability compared to any of the drugs on their own under normoxic conditions. Under hypoxic conditions, the combination of 2DG and 1μg/ml Dox, as well as Dox alone, lost its effectiveness. Encouragingly though, ^19^FDG together with 1μg/ml Dox maintained its efficacy, significantly decreasing cell viability compared to Dox, ^19^FDG, or 2DG alone and the combination of 2DG with 1μg/ml Dox.

### ^19^FDG maintains its ability to sensitize HeLa cells to doxorubicin and induce apoptosis in hypoxia

**Fig 3:**
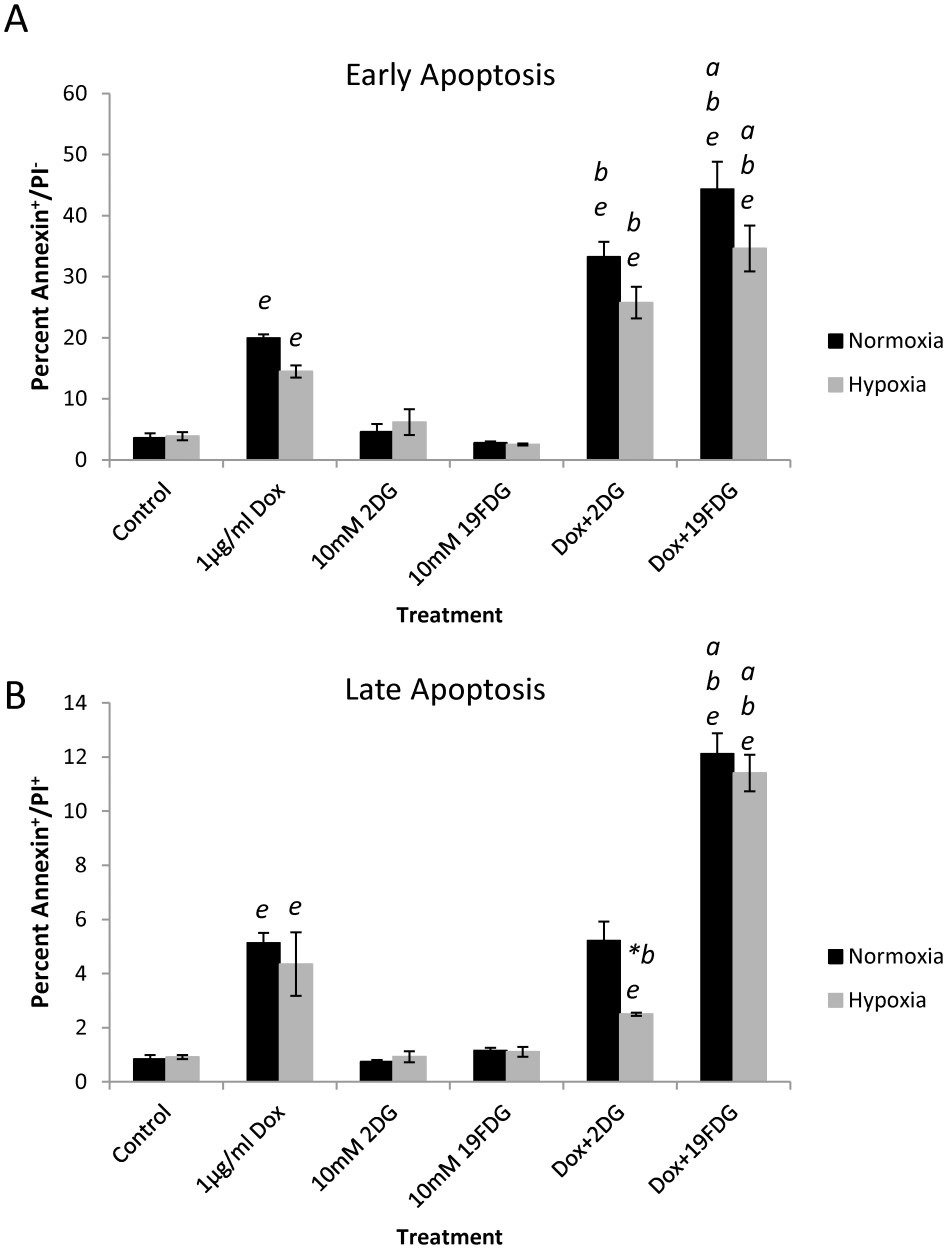
Measurement of HeLa cell death following combination treatment. HeLa cells were treated under normoxic or hypoxic (1% O_2_) conditions with either Dox, non-radioactive 2-deoxy-2-fluoro-D-glucose (^19^FDG)/2-deoxy-D-glucose (2DG), or a combination of the two for 12 hours. Flow cytometry was performed to observe annexin V and propidium iodide staining. Panel A shows annexin positive/propidium iodide negative (Annexin^+^/PI^-^) results representing early apoptosis. Panel B shows annexin positive/propidium iodide positive (Annexin^+^/PI^+^) results representing late apoptosis. n = 6 for control and Dox samples, n = 3 for all other treatments. *a*, denotes significance from Dox+2DG, *b*, denotes significance from Dox alone. *b, denotes a significant decrease in effect from Dox alone. *e*, denotes significance from control. p≤0.05.

In order to ensure that treatment with the glucose analogs was not only slowing cell growth, but also resulting in cell death, several stages of cell death were observed (Fig 3). Both 2DG and ^19^FDG in combination with 1μg/ml Dox significantly increased the percentage of cells in early apoptosis compared to any of the treatments on their own. In the case of late apoptosis/necrosis, the combination of 2DG and Dox has no effect above Dox alone, and there is a significant loss in effect of this combination under hypoxic conditions. ^19^FDG however maintains its ability to induce cell death under hypoxic conditions when in combination with Dox. At all stages of cell death, the combination of ^19^FDG and Dox exhibited a higher proportion of apoptotic and or necrotic cells, compared to 2DG and Dox together.

### ^19^FDG successfully inhibits glucose uptake in MDA-MB-231 tumour xenografts

**Fig 4:**
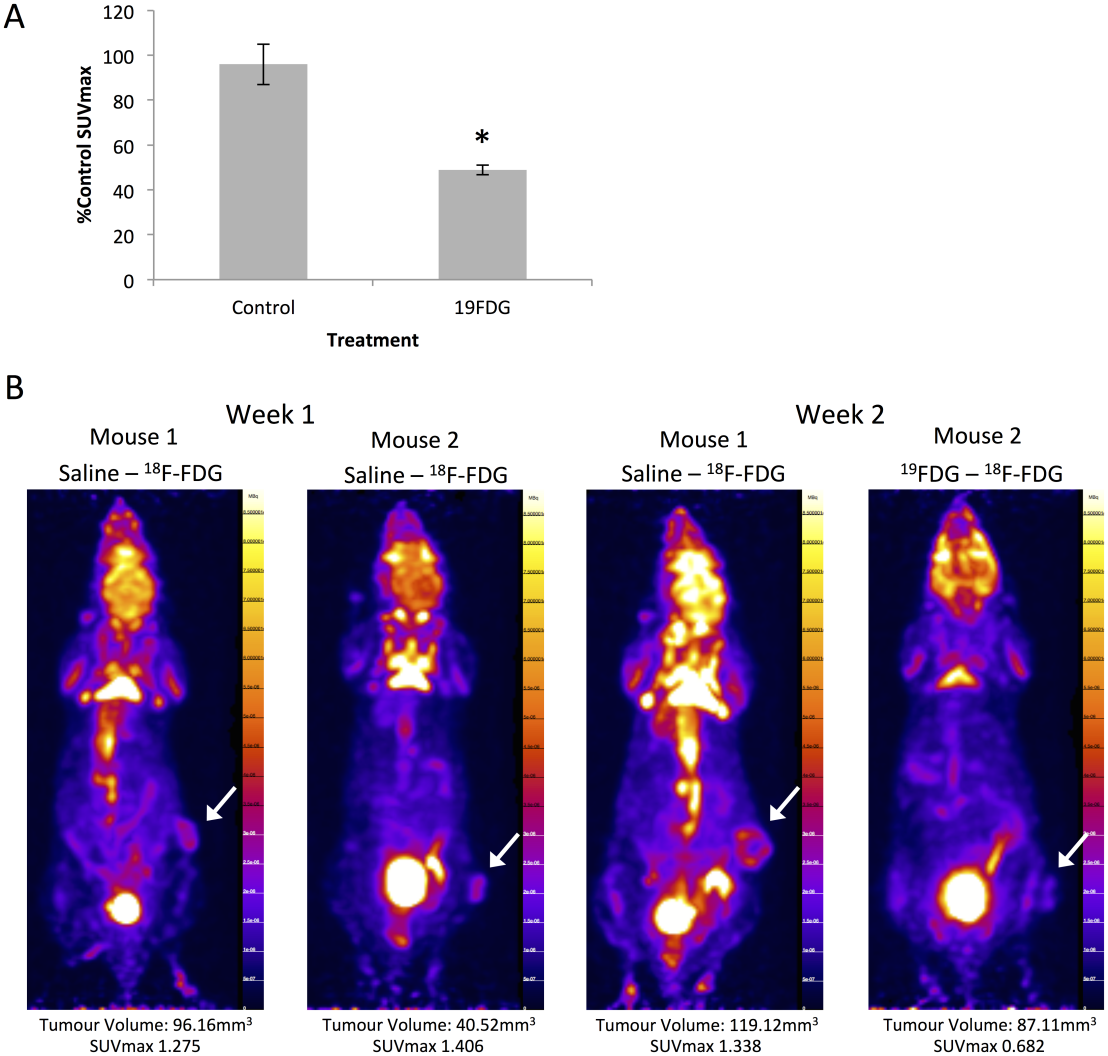
Glucose uptake inhibition in MDA-MB-231 tumours *in vivo*. A solution of five million MDA-MB-231 cells in serum free media was injected into the right leg/flank area of athymic nude mice. Tumours were allowed to grow for 3–4 weeks before mice were imaged. Prior to imaging, mice were fasted 5 hours. Once tumours reached an SUVmax of at least 1 (deemed “week 1”), mice were placed into either the control group or the non-radioactive 2-deoxy-2-fluoro-D-glucose (^19^FDG) group. The ^19^FDG group received 220mg/kg ^19^FDG 2 hours prior to ^18^F-FDG. Intraperitoneal saline injections were used as an injection control in the control group mice. 20μCi ^18^F-FDG was injected into the intraperitoneal cavity of all mice 1 hour prior to imaging with the microPET. The second round of imaging took place one week after the first. Panel A shows SUVmax results from week 2 as a percentage of the week 1 imaging results. n = 2–3, * denotes significance from control mice, p≤0.05. Panel B shows representative reconstructed microPET images of mice following week one and week two of treatment. The white arrow highlights the tumour location.

Once the success of ^19^FDG was determined *in vitro*, we next performed PET imaging studies *in vivo*, using a tumour xenograft model. PET imaging studies performed in our lab revealed that ^19^FDG given 2 hours prior to ^18^F-FDG allowed for the best inhibition of glucose uptake in the tumour, an important finding for designing combination treatment studies. SUVmax values revealed that while the control group glucose uptake remained unchanged between the two imaging days (one week apart), the ^19^FDG group exhibited a significant decrease in SUVmax following ^19^FDG administration in week 2 (Fig 4).

## Discussion

Adjuvant treatment of tumours through combining the inhibition of glycolysis with standard of care has the potential to improve therapeutic outcomes. Progress has been made regarding the general efficacy of glycolytic inhibition as a cancer treatment procedure, however the optimal method of inhibition is still unknown. While 2DG has shown some promising results *in vitro* and *in vivo* [25–31], its use has been impeded by several limitations [30, 32] (Clinicaltrial.gov Identifiers: NCT00247403 and NCT00633087), highlighting the need for a different option. ^19^FDG is one such option, as it is able to successfully inhibit glycolysis [34] without some of the limitations of 2DG, such as losing its effectiveness in hypoxia. ^19^FDG also acts similarly to 2DG, becoming trapped in the cell through phosphorylation and inhibiting further uptake of glucose. Specifically, the fluorination of ^19^FDG effectively traps the molecule as ^19^FDG-6-phosphate. This causes an unfavourable concentration gradient across the cell membrane, resulting in decreased glucose uptake. This also results in the inability of the cell to complete the pentose phosphate pathway, disabling the cell from scavenging ROS [12]. In this study, we show that ^19^FDG is superior to 2DG with respect to its ability to block glycolysis.^19^FDG also maintains its function under hypoxic conditions, and works synergistically with Dox to cause a more potent effect than either ^19^FDG or Dox alone with respect to decreasing cell viability and increasing apoptosis.

While the most relevant findings in this study involve the combination of ^19^FDG and Dox, ^19^FDG on its own did show a significant improvement in inhibiting lactate production compared to 2DG *in vitro*. This could be explained by differential affinities between the compounds for the GLUT1 transporter. Gorovits et al. [46] found that the GLUT1 Km value for glucose is 1-2mM, while its Km for 2DG is 6.9mM [47]. Of note, ^19^FDG is more structurally similar to glucose than 2DG is to glucose [48], suggesting that the Km for ^19^FDG may be similar to glucose, resulting in GLUT1 having a higher affinity for ^19^FDG than for 2DG. These structural similarities between ^19^FDG and glucose likely contribute to properties of ^19^FDG that make it a better analog of glucose than 2DG, and could explain the observed improved success.

Dox is currently used for treating many types of cancers including both solid tumours and blood cancers. While it has therapeutic benefits, Dox also has serious side effects, at times causing cardiac failure [49, 50] in a dose-dependent manner. We show here that the combination of ^19^FDG with Dox results in a more potent effect with respect to cell viability and apoptosis compared to Dox alone, suggesting that there might be a sensitization effect. It may also be that the combined effect from increased ROS following Dox treatment and the inability to scavenge ROS following inhibition of glycolysis is enough to surpass the protective mechanisms built up in these cancerous cells. Combining this drug with ^19^FDG may therefore allow for a lower administrative dose of the Dox, which will decrease the risk of cardiotoxicity and other side effects. It is hypothesized that Dox can affect cancerous cells by 1) generating free radicals, which damage DNA, and 2) intercalating into the DNA, disrupting topoisomerase II-mediated DNA repair [51]. The extent of free radical generation and DNA intercalation is likely dependent on dose with lower doses, such as those used in the current study, making DNA intercalation more likely [52]. Previous studies have also shown an enhancement of anti-tumour activity from topoisomerase II inhibitors in the presence of 2DG [27, 53], possibly from an additional promotion of oxidative stress through glucose deprivation [54]. Our results show that this effect is somewhat diminished under hypoxic conditions with respect to 2DG (in line with previous studies), but that the combination of Dox and ^19^FDG remains effective in its anti-tumour action, significantly increasing apoptosis and decreasing cell viability compared to 2DG with Dox. This ability is vital for a successful cancer treatment method as hypoxic areas in tumours are typically the most difficult to treat.

Although the cell culture results are favourable for the use of ^19^FDG, there is a general lack of research involving ^19^FDG and *in vivo* models. Indeed, if ^19^FDG were to be used as a potential adjuvant therapy for treating aggressive tumours in combination with other chemotherapies, a technique that could determine tumour glucose uptake at various doses of ^19^FDG would be invaluable to help to guide dosing regimens and avoid side effects. Using doses of ^19^FDG that avoid glycolytic inhibition in hyperglycolytic non-cancerous tissues, such as the brain or heart, would be critical for success. In this study, we have used PET and ^18^F-FDG to measure the ability of ^19^FDG to inhibit glucose uptake, and monitor this inhibition, in tumour xenografts. Our data reveal that at a dose of 220mg/kg ^19^FDG, a 50% inhibition of breast cancer tumour glucose uptake is observed, an effect that was also seen in glycolytically active non-tumour tissues such as the heart and the brain (S1 Table). The effect on glucose uptake in the tumour is transient with an optimal effect at approximately 2 hours, and almost no effect seen at 8 hours post-treatment (S1 Fig), suggesting that the ^19^FDG treatment will not have a lasting detrimental effect or build up in other parts of the body, a critical aspect for future human studies. While these results are promising, further studies are needed to determine if the use of ^19^FDG in combination with other compounds such as Dox is efficacious *in vivo*. This is also important given previous literature showing false-positive PET results due to chemotherapy-induced inflammation [55–57].

While 2DG has been shown to be effective in some conditions, the present study has shown the ability of ^19^FDG to surpass any of the positive effects of 2DG. We also show here that ^19^FDG in combination with Dox maintains its effectiveness under hypoxic conditions, something that is crucial for treatment success *in vivo*. Further, PET imaging has been a useful tool to evaluate the effects of ^19^FDG as a competitive inhibitor of ^18^F-FDG uptake *in vivo* and will be a critical part of future studies, guiding dosing regimens to optimize therapeutic effects while limiting side effects. Taken together, our results suggest ^19^FDG demonstrates the potential to be developed as part of an adjuvant therapy for the treatment of tumours.

## Supporting information

S1 Fig ^19^FDG timecourse experiment. Mice were injected in week 1 with saline as a control, and ^**18**^F-FDG uptake was measured using the microPET. Mice were then treated in week 2 with ^**19**^FDG for either 2 hours, 4 hours, or 8 hours prior to imaging, and ^**18**^F-FDG uptake was measured. Results are week 2 SUVmax presented as an average percentage of week one control SUVmax. n = 1–3.
(TIFF)

S1 Table Brain and heart ^18^F-FDG uptake following administration of either saline as control or 220mg/kg ^19^FDG IP. Mice were injected in week 1 with saline as a vehicle (saline), and ^**18**^F-FDG uptake was measured using the microPET. Mice were then separated into saline and ^**19**^FDG groups and ^**18**^F-FDG uptake was measured a second time, one week later (week 2). Results are presented as week two SUVmax presented as an average percentage of week one saline SUVmax. n = 2–3.*denotes significance from saline treated mice following a t-test. p≤0.05.
(TIFF)
