# Supplementary figures and images for: Non-radioactive 2-deoxy-2-fluoro-D-glucose inhibits glucose uptake in xenograft tumours and sensitizes HeLa cells to doxorubicin in vitro
Source: PLoS One. 2017 Nov 2;12(11):e0187584. doi: 10.1371/journal.pone.0187584 (PMC5667878; doi:10.1371/journal.pone.0187584)

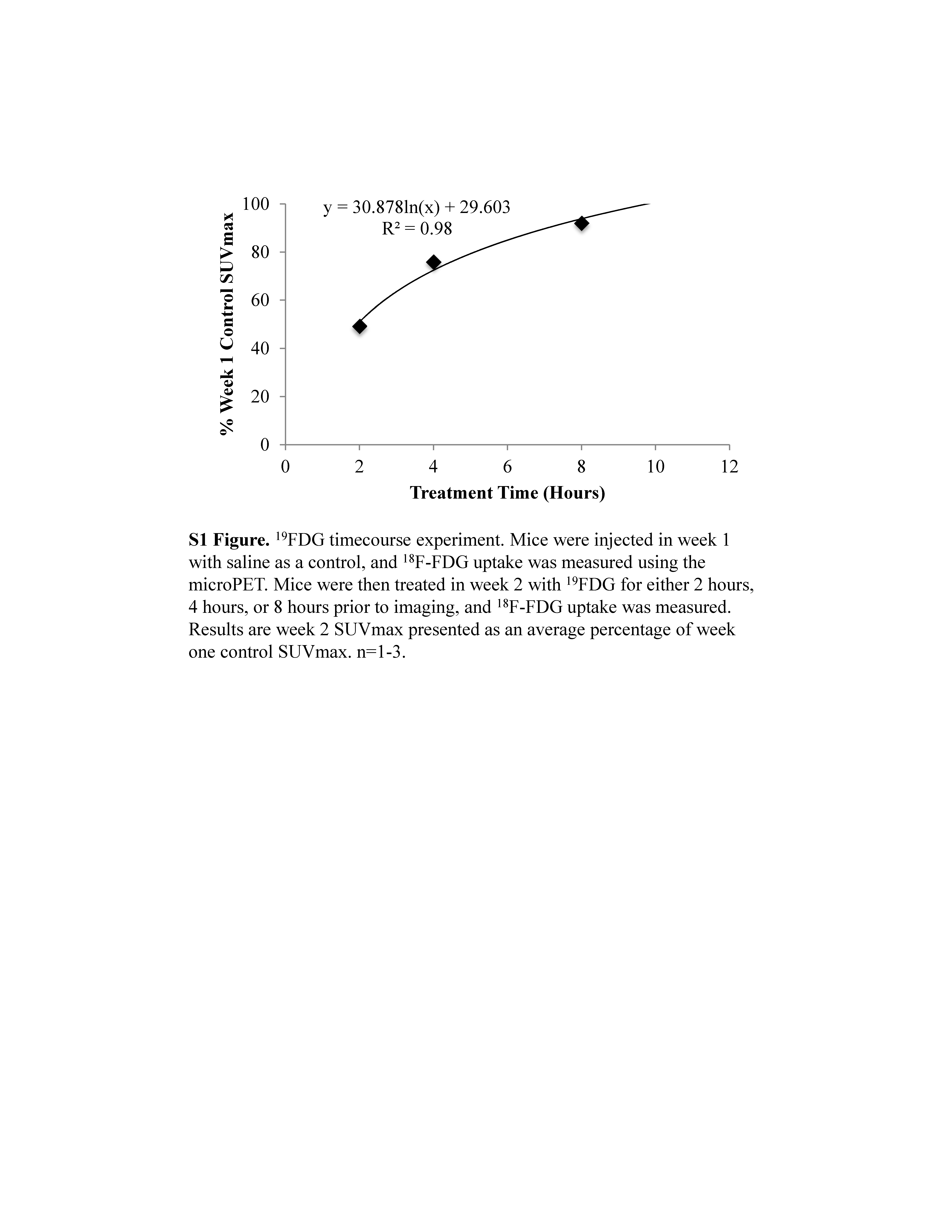

Supplement: S1 Fig — Mice were injected in week 1 with saline as a control, and 18F-FDG uptake was measured using the microPET. Mice were then treated in week 2 with 19FDG for either 2 hours, 4 hours, or 8 hours prior to imaging, and 18F-FDG uptake was measured. Results are week 2 SUVmax presented as an average percentage of week one control SUVmax. n = 1–3. (TIFF) [file pone.0187584.s001.tiff]
